# Executive functions scale for university students: UEF-1

**DOI:** 10.3389/fpsyg.2023.1192555

**Published:** 2023-07-13

**Authors:** Carlos Ramos-Galarza, Valentina Ramos, Milenko Del Valle, Nancy Lepe-Martínez, Jorge Cruz-Cárdenas, Pamela Acosta-Rodas, Mónica Bolaños-Pasquel

**Affiliations:** ^1^Facultad de Psicología, Pontificia Universidad Católica del Ecuador, Quito, Ecuador; ^2^Centro de Mecatrónica y Sistemas Interactivos MIST, Facultad de Psicología, Universidad Tecnológica Indoamérica, Quito, Ecuador; ^3^Grupo de Investigación en Sistemas de Información, Gestión de la Tecnología e Innovación, Escuela Politécnica Nacional, Quito, Ecuador; ^4^Universidad de las Américas, Quito, Ecuador; ^5^Facutlad de Ciencias Sociales, Artes y Humanidades, Universidad de Antofagasta, Antofagasta, Chile; ^6^Facutlad de Ciencias de la Educacion, Departamento de Diversidad e Inclusividad Educativa, Talca, Chile; ^7^Centro de Investigación ESTec, Universidad Tecnológica Indoamérica, Quito, Ecuador

**Keywords:** executive functions, university students, learning, behavior, academic performance

## Abstract

**Introduction:**

Executive functions are a set of mental abilities that allow human beings to consciously regulate their behavior and, in a university setting, will have a significant impact on student success during professional training.

**Objective:**

To develop a scale to assess executive functions in a university setting.

**Method:**

Using a sample of 1,373 university students from Chile (663) and Ecuador (710) between 17 and 33 years old (*M*_age_ = 20.53, *SD* = 2.34). A study was carried out to analyze the psychometric properties of the instrument using a reliability and validity analysis for a scale that assesses executive functions: conscious monitoring of responsibilities, supervisory attentional system, conscious regulation of behavior, verification of behavior to learn, decision making, conscious regulation of emotions, and management of elements to solve tasks.

**Results:**

Adequate internal consistency parameters were found between *α* = 0.71 and 0.85. The seven executive functions proposed on the scale correlated proportionally between *r* = 0.42 and 0.62. In the confirmatory factor analysis, good fit indices were obtained in the model of the seven executive functions *x^2^_(413)_* = 1649.14, *p* = <0.001, *CFI* = 0.91, *SRMR* = 0.04 and *RMSEA* = 0.04.

**Discussion:**

The research carried out reaches its conclusion stating that the scale that was developed has the psychometric properties to assess executive functions in the Latin American setting. The results regarding previous research and the contribution made in the line of research of executive functions are discussed.

## 1. Introduction

Executive functions are a set of cognitive abilities that allow human beings to consciously regulate their behavior (Ramos-Galarza et al., 2017a,b). The main executive functions that have been reported are the organization of elements for tasks, conscious monitoring of behavior and responsibilities that must be executed, deliberate regulation of emotions, decision making, ability to attend voluntarily, verification of acts and tasks, to comply, regulate impulses, among other higher-order mental abilities (Ramos-Galarza et al., 2022; Doebel and Lillard, 2023). Classic authors of executive functions such as Lezak (1995), Baddeley and Hitch (1974), Diamond (2013), Jurado and Rosselli (2007), Anderson et al. (2001), Goldberg (2002), and Luria (1980) state that these higher order mental abilities allow human beings to plan, regulate and direct their behavior consciously, with an efficient and creative style towards a goal, respecting the different socially established parameters.

In relation to explanatory models of executive functions, it is a subject that is still under theoretical construction, since there are authors who propose single or multifactorial models. For example, Barkley (1997) proposes that there is a central executive function (inhibitory control) that controls the conscious regulation of behavior, Norman and Shallice (1986) propose the existence of a supervisory attentional system in which executive functions are activated in novel situations, Gioia et al. (2002) propose that executive functions interact in three major factors: metacognition, emotional regulation and behavioral regulation.

Diverse research has presented the role of executive functions in the setting of human daily life (Józsa et al., 2022). For example, the role of the environment as a stimulator of executive functions has been identified, where social, economic (Hilton et al., 2022; Murtha et al., 2023), health aspects (Stinchcombe et al., 2023; Wang et al., 2023) or sports practices (Jun, 2023) play a determining factor in the development. In addition, executive functions have been identified in relation to the success of human beings in educational settings (Stark and Lindo, 2022; Heitzer et al., 2023; Kolovelonis et al., 2023), regarding the reduction of problems such as stress and anxiety (Anderson and Marino, 2022). And the research highlights the importance of studying executive functions for the benefit of conscious regulation of individual behavior (Ramos-Galarza et al., 2020).

In the university setting, which is the focus of this research, executive functions such as working memory and conscious supervision of behavior are identified as a decisive role in areas such as academic performance in higher education, since students at this level must be aware of each of their actions all the time and have information in their minds to satisfactorily meet the challenges they encounter during their professional training (Ramos-Galarza et al., 2020).

Other executive functions identify that the factors for the self-regulation processes for university student learning are problem solving, planning, development and implementation of strategies, verbal reasoning, sequencing, attentional system, cognitive flexibility, and impulse inhibition. Therefore, reaching the optimal level of these executive functions will depend on, to a large extent, the success of the conscious learning that university students manifest, their permanence, and adequate academic performance (Pinochet-Quiroz et al., 2022).

Other areas where executive functions have been identified and studied, that help university students, in addition to academic performance, are the relationship of these mental abilities with social interaction and how they comply with established norms (Hilton et al., 2022), how they express themselves adequately emotionally in the relationships they establish with their peers (Jiang et al., 2022), how they plan activities such as trips or avoid obstacles that negatively influence their lives (Bocchi et al., 2022), their quality of sleep and levels of creativity (Guo et al., 2022), health levels (Liu et al., 2022), physical activity planning (Meng, 2022), social network management and eating habits (Zhonghua and Mingde, 2022).

Regarding the evaluation of executive functions, there are three types of procedures: (a) tests created to evaluate executive functions, (b) non-specific tests, created for other purposes but that contribute at a clinical level to assess executive functions, and (c) delayed observation tests of self-report and hetero-report behavior, which are a scale or questionnaire made to assess the executive function from a subject’s behavioral perspective (García-Gómez, 2015; Ramos-Galarza et al., 2018; Kusi-Mensah et al., 2022). In the case of the present research, it is in the team’s interest to propose a self-report scale of the university student’s behavior, which allows to identify how their executive functions are in the daily and everyday activities performed by the student. The development of this scale is justified because the research interest in executive functions has focused more on child development, adolescent and adult population, however, the methods of measurement exclusively for university students is a topic still under development in this line of research, for this reason, the contribution of this study lies in the creation and validation of a new instrument that is beneficial in a population still under study and that needs measurement instruments specific to their reality (Fenn et al., 2020).

This research is part of the latest procedures for the neuropsychological evaluation of executive functions, its purpose is to present a means to carry out the behavioral observation of university students that helps measure these higher mental abilities and to understand their role in order to standardize student learning behaviors. In Latin America, it is essential to develop means to measure executive functions that allow studies to be carried out with university students (Pedrero-Pérez et al., 2016; Ramos-Galarza et al., 2018, 2019). In addition to having an adequate means to assess executive functions in Latin America, the scale proposed herein will be free of charge so that it can be used without restrictions in future studies at the university level.

## 2. Methods

### 2.1. Participants

We worked with a Latin American sample of 1,373 university students between 17 and 33 years old (*M*_age_ = 20.53, *SD* = 2.34) from Chile and Ecuador. In Chile, there were 663 (*M_age_* = 20.23, *SD* = 2.42), in relation to gender, 308 (46.46%) participants were women and 355 (53.54%) men. Regarding the type, 65 participants (9.80%) studied in a private, 188 (28.36%) state, 272 (41.03%) municipal, and 138 (20.81%) a sponsored institution. All the participants belonged to the Chilean university system. In Ecuador, we worked with a sample of 710 participants (*M_age_* = 20.80, *SD* = 2.21), in terms of gender, 465 (65.49%) were women, and 245 (34.51%) men. All the participants belonged to the Ecuadorian university system. Concerning the type, 521 (73.38%) studied in a private, 140 (19.72%) state, 47 (6.62%) municipal, and 2 (0.28%) in a sponsored university. None of the participants had a history of psychological disorders, drug use or any type of health condition that could influence their executive functioning.

### 2.2. Measuring instruments

The executive functions scale for a university setting is configured with 31 items and measures 7 executive functions (UEF-1): Conscious monitoring of responsibilities (UEF1: items 2, 8, 9, 15, and 27), Supervisory attention system (UEF2: items 10, 14, 22, 28, and 13), Conscious regulation of behavior (UEF3: items 3, 11, 16, 17, 18, and 19), Verification of behavior to learn (UEF4: items 20, 23, 24, and 30), Decision making (UEF5: items 5, 12, and 21), Conscious regulation of emotions (UEF6: items 4, 25, 29, and 31) and Management of elements to solve tasks (UEF7: items 1, 6, 7, and 26). The full scale can be seen in Annex 1.

The construction of this instrument is based on the need to have an instrument that encompasses the daily life situations faced by university students, which are not considered in the instruments developed to measure executive functions for the adult population in general. The process followed was based on the proposal of Fenn et al. (2020):

- Item construction: a list of items was proposed based on classical theories of executive functions such as Lezak, Baddeley, Diamond, Anderson and Luria adapted to the university context. The items were proposed by the authors of the research, who are experts in university education and research on executive functions.
- Format of the instrument: the format was a quantitative Likert-type question with 5 response options that allow assessing the level of executive function of the university student. Each item presents statements of daily life situations that the student may encounter.
- Linguistic analysis: the items were analyzed in cognitive interviews with university students, who allowed us to identify the content adjustments necessary to present the instrument to the participants in each country.
- Expert judgment: each item was reviewed by expert researchers and they contributed to ensure that the content measured the proposed executive function.
- Pilot study: once the instrument was in its best presentation, a pilot study was applied in Chile and Ecuador. This process allowed us to identify possible problems with the items and to configure the final version of the instrument for its application.
- Quantitative analysis: subsequently, reliability and validity values were analyzed, which are presented in the results section.
- Calculation of statistical power: to perform this procedure, 15 participants for each item applied were configured, giving a sample of 285 participants as a minimum necessary for the execution of the study; however, in this study we have a sample of 1,373 university students.

### 2.3. Data analysis plan

For this research, the following statistical analyzes were applied: (a) descriptive statistics to present sociodemographic data and data of central tendency and dispersion of executive functions, (b) Cronbach’s alpha and McDonald’s omega to analyze the reliability of the subscales, (c) correlation to identify the relationship between the items of each scale and the subscales of the instrument, (d) a chi-square to analyze the relationship between executive functions and sociodemographic variables, and (e) confirmatory factor analysis to identify the validity of the model of executive functions. All the analyzes were carried out in the SPSS version 28 and AMOS version 28 software.

### 2.4. Procedure

This research began with the approval of the Ethics for Human Beings Committee of the Pontificia Universidad Católica del Ecuador. Subsequently, the following steps were carried out: (1) the request for authorization to conduct the research in Chilean and Ecuadorian universities, (2) the construction of the measuring instrument by the team of researchers from Chile and Ecuador, (3) the obtention of the informed consent for voluntary participation, (4) the implementation of cognitive interviews as part of the instrument content validation process, (5) the implementation of a pilot study with a group of university students, (6) the observations to improve the instruments, (7) the completion of the measuring instruments in a massive way in universities of the two countries, (8) the validation of the instruments and elimination of around 100 questionnaires that presented errors or where the voluntary participation consent form had not been signed and (9) the drafting of this research report.

## 3. Results

### 3.1. Reliability of executive functions

The reliability was calculated according to Cronbach’s Alpha and McDonald’s Omega, where it was found that the subscales that assess the different executive functions have an adequate parameter in their internal consistency. Supervisory attentional system *α* = 0.85 and *Ω* = 0.82, conscious regulation of emotions *α* = 0.82 and *Ω* = 0.75, conscious monitoring of responsibilities *α* = 0.80 and *Ω* = 0.75, verification of behavior to learn *α* = 0.71 and *Ω* = 0.65, management of elements to solve tasks *α* = 0.76 and *Ω* = 0.72, decision making *α* = 0.71 and *Ω* = 0.65, conscious regulation of behavior *α* = 0.76 and *Ω* = 0.74.

In the correlations between each item that made up each executive function, significant and directly proportional correlations were found, in medium and large magnitudes (*r* = 0.41 and 0.70). No items were found that generate any negative aspect with the reliability of the scale, therefore, it was not necessary to eliminate any of them.

### 3.2. Descriptive statistics of executive functions

Table 1 shows the values corresponding to central tendency and dispersion for each of the executive functions proposed for the scale to be used in a university setting.

**Table 1:** Descriptive statistics.

	Min.	Max.	M	SD
Supervisory attentional system	12.00	40.00	29.96	5.16
Deliberate regulation of emotion	5.00	25.00	18.49	3.89
Conscious monitoring of responsibilities	16.00	35.00	30.37	3.48
Conduct verification	9.00	25.00	20.81	2.94
Organization of elements for tasks	7.00	20.00	16.56	2.82
Conscious regulation of behavior	17.00	40.00	32.74	4.22
Decision making	7.00	25.00	20.06	2.93

### 3.3. Correlation between executive functions

Once each of the executive functions was configured, the correlation between them was analyzed. It was found that there are statistically significant correlations of medium and large magnitude between the executive functions (Table 2).

**Table 2:** Analysis of correlations.

	1	2	3	4	5	6	7
1. Supervisory attentional system	1						
2. Deliberate regulation of emotion	0.42^**^						
	<0.001	1					
3. Conscious monitoring of responsibilities	0.62^**^	0.30^**^					
	<0.001	<0.001	1				
4. Verification of conduct	0.59^**^	0.28^**^	0.62^**^				
	<0.001	<0.001	<0.001	1			
5. Organization of elements for tasks	0.46^**^	0.27^**^	0.40^**^	0.43^**^			
	<0.001	<0.001	<0.001	<0.001	1		
6. Conscious regulation of behavior	0.56^**^	0.54^**^	0.52^**^	0.55^**^	0.43^**^		
	<0.001	<0.001	<0.001	<0.001	<0.001	1	
7. Decision making	0.57^**^ <0.001	0.49^**^ <0.001	0.56^**^ <0.001	47 <0.001	0.35 < 0.001	0.49^**^ <0.001	1

^**^p < .001

### 3.4. Association of executive functions with sociodemographic variables

A statistical analysis was carried out to identify the association of each executive function with the different sociodemographic variables of the sample. Table 3 shows the results found.

**Table 3:** Association analysis.

	Gender	Age	Semester	Nationality	Institution
1. Supervisory attentional system	*X^2^* = 29.18 *p* = 0.40	*X^2^* = 417.32 *p* = 0.53	*X^2^* = 357.84 *p* = 0.02	*X^2^* = 42.50 *p* = 0.04	*X^2^* = 100.01 *p* = 0.11
2. Deliberate regulation of emotion	*X^2^* = 31.99 *p* = 0.04	*X^2^* = 243.31 *p* = 0.99	*X^2^* = 197.08 *p* = 0.86	*X^2^* = 55.64 *p* = <0.001	*X^2^* = 80.23*p* = 0.04
3. Conscious monitoring of responsibilities	*X^2^* = 26.43 *p* = 0.07	*X^2^* = 236.21*p* = 0.80	*X^2^* = 216.77 *p* = 0.07	*X^2^* = 32.25 *p* = 0.01	*X^2^* = 46.86 *p* = 0.64
4. Verification of behavior to learn	*X^2^* = 28.13 *p* = 0.03	*X^2^* = 215.79 *p* = 0.86	*X^2^* = 213.06 *p* = 0.03	*X^2^* = 34.69 *p* = 0.01	*X^2^* = 48.77 *p* = 0.44
5. Management of elements to solve tasks	*X^2^* = 14.76 *p* = 0.32	*X^2^* = 162.47 *p* = 0.98	*X^2^* = 175.70 *p* = 0.03	*X^2^* = 40.97 *p* = <0.001	*X^2^* = 62.40 *p* = 0.01
6. Conscious regulation of behavior	*X^2^* = 23.67 *p* = 0.26	*X^2^* = 301.58 *p* = 0.46	*X^2^* = 255.11 *p* = 0.05	*X^2^* = 86.34 *p* = <0.001	*X^2^* = 80.27 *p* = 0.04
7. Decision making	*X^2^* = 21.81 *p* = 0.19	*X^2^* = 240.97 *p* = 0.72	*X^2^* = 231.85*p* = 0.01	*X^2^* = 18.50 *p* = 0.35	*X^2^* = 43.50 *p* = 0.76

### 3.5. Confirmatory factor analysis

**Figure 1:**
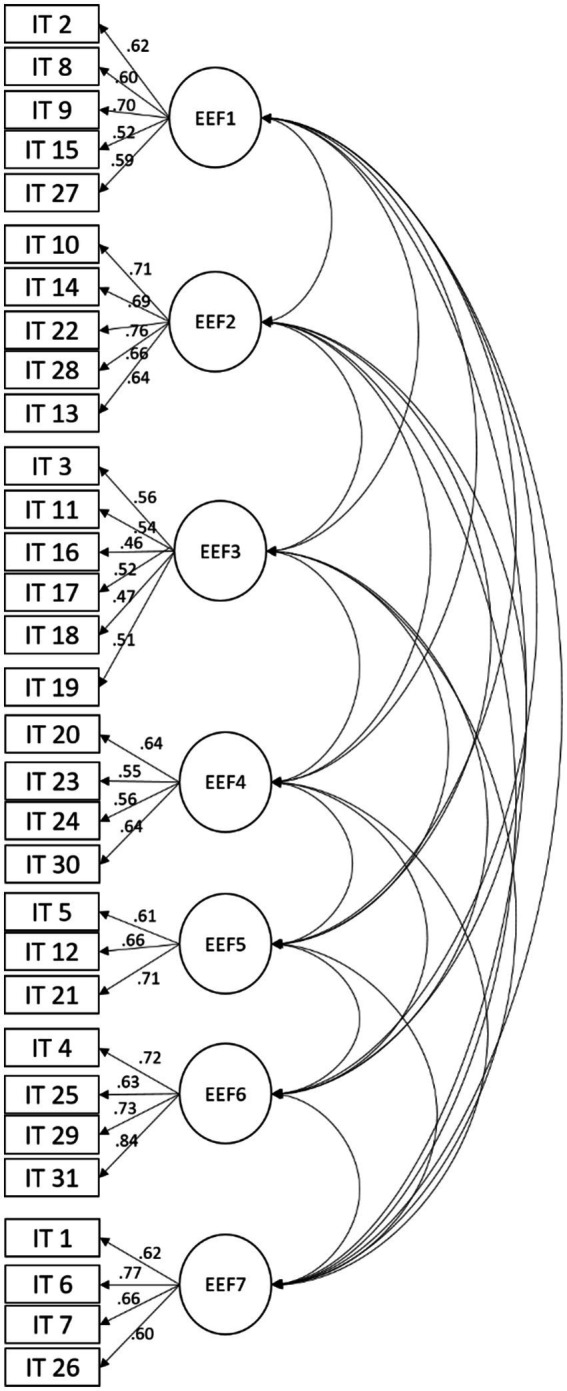
Model of executive functions in a university setting.

The factorial organization of the scale was analyzed, and a model was proposed with the 7 key executive functions in a university setting: Conscious monitoring of responsibilities (UEF1), Supervisory attention system (UEF2), Conscious regulation of behavior (UEF3), Verification of behavior to learn (UEF4), Decision making (UEF5), Conscious regulation of emotions (UEF6) and Management of elements to solve tasks (UEF7). Figure 1 shows the proposed model.

In the statistical analysis, it was found that there is an adequate adjustment of the proposed model with indicators: *x^2^*_(413)_ = 1649.14, *p* = <0.001, *CFI* = 0.91, *SRMR* = 0.04 and *RMSEA* = 0.04. Table 4 shows the regression weights of the proposed model.

**Table 4:** Regression weights of the proposed model.

			Estimate	S.E.	C.R.
EF37	<−--	UEF7	1.00		
EF7	<−--	UEF7	1.13	0.06	17.91
EF6	<−--	UEF7	1.36	0.07	19.27
EF1	<−--	UEF7	0.93	0.05	17.15
EF18	<−--	UEF1	0.67	0.04	15.18
EF9	<−--	UEF1	1.01	0.05	18.87
EF8	<−--	UEF1	0.96	0.06	16.90
EF22	<−--	UEF3	0.85	0.06	13.49
EF21	<−--	UEF3	0.98	0.07	14.62
EF20	<−--	UEF3	0.77	0.06	13.31
EF42	<−--	UEF6	1.06	0.04	27.61
EF40	<−--	UEF6	1.00		
EF34	<−--	UEF6	0.81	0.04	21.76
EF4	<−--	UEF6	0.83	0.03	24.52
EF26	<−--	UEF5	1.00		
EF13	<−--	UEF5	0.91	0.05	19.71
EF5	<−--	UEF5	0.82	0.04	18.62
EF27	<−--	UEF2	1.09	0.05	22.71
EF41	<−--	UEF4	1.00		
EF33	<−--	UEF4	0.86	0.05	16.64
EF31	<−--	UEF4	0.92	0.06	16.10
EF24	<−--	UEF4	1.00	0.06	18.20
EF38	<−--	UEF1	1.00		
EF2	<−--	UEF1	0.94	0.05	17.46
EF17	<−--	UEF2	1.02	0.05	21.20
EF10	<−--	UEF2	1.12	0.05	21.67
EF39	<−--	UEF2	1.13	0.06	20.44
EF15	<−--	UEF2	1.00		
EF3	<−--	UEF3	1.00		
EF11	<−--	UEF3	1.33	0.09	14.94
EF23	<−--	UEF3	0.98	0.07	14.72

**Figure 2:**
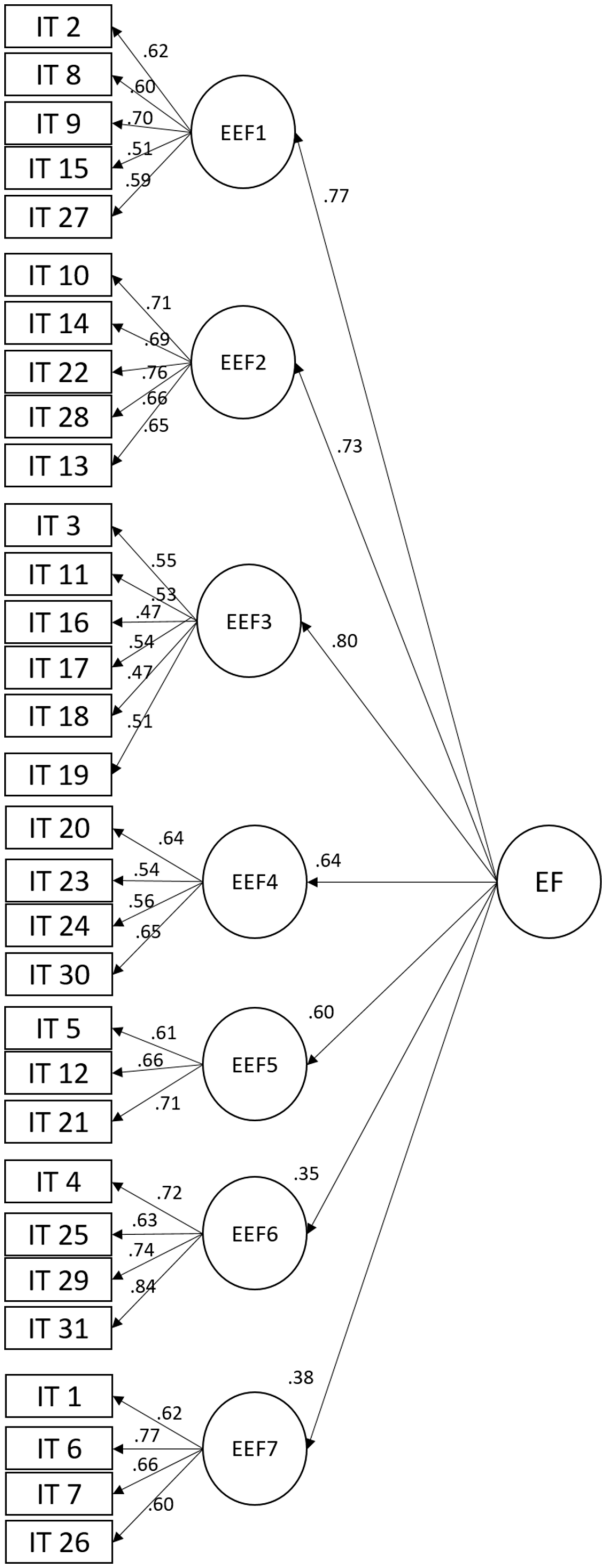
Second-order executive functions model.

Second, in the confirmatory factor analysis, a second order model was tested, taking into consideration a central factor of executive functions, where it was found that it does not present an acceptable fit *x^2^_(427)_* = 1887.32, *p* = <0.001, *CFI* = 0.89, *SRMR* = 0.05 y *RMSEA* = 0.05. Figure 2 shows the proposed model.

## 4. Discussion

Regarding this research, herein is a report about a study that proposes a scale to assess executive functions in a university setting. In the proposed instrument, the executive functions considered are: (a) organization of elements for tasks, (b) conscious monitoring of responsibilities, (c) conscious regulation of behavior, (d) deliberate regulation of emotions, (e) taking decisions, (f) supervisory attention system and (g) verification of compliance with objectives.

The aforementioned executive functions have been taken into consideration based on classical theories that propose that the role of the mental abilities of the frontal lobe can successfully face the challenge of higher education, as exampled by Luria (1973, 1980) who speaks of the importance of planning, regulating and verifying cognitive and behavioral activity, Damasio (1994) who presents the role of decision-making in the daily life of the human being, or Lezak (1995) who affirms the importance of executive functions to meet objectives within what’s socially established.

The importance of studying executive functions in a university setting lies in the need to develop competences in students so that they can consciously regulate their behavior to improve their academic performance (Ramos-Galarza et al., 2016, 2017a,b). To this end, the scale proposed in this article will allow new research to be carried out that will benefit university students and their performance which in turn will favor their professional training and, therefore, have a positive impact on society (Ramos-Galarza and Pérez-Salas, 2016).

The results of this research are consistent with previous studies that have found adequate psychometric properties to assess executive functions in various settings, for example, the study carried out by Korzeniowski and Ison (2019) who developed a scale in a school setting with executive functions: attention regulation, metacognition, inhibitory regulation, organization, planning, and flexibility. In another study, that of García et al. (2018), a scale was analyzed to assess executive functions in a family setting and took into consideration inhibition, hyperactivity, emotion regulation, concentration, focus, organization, working memory, and flexibility as executive functions.

Another research that is consistent with the study reported in this article is the report by Parhoon et al. (2022), Arruda et al. (2022), Castagna et al. (2019), Ramos-Galarza et al. (2019), and Bausela (2019) who highlight the importance of this type of executive function scale to analyze the symptoms of attention deficit and hyperactivity disorder to identify developmental disorders and to analyze the role of executive functions in the educational setting.

As seen in previous research and in what has been reported in this article, the study of executive functions is vital for university students to be able to adequately regulate their behavior in favor of learning (Cushman et al., 2022). For this reason, it is essential to continue deepening research to explore executive functions such as the organization of elements to solve tasks, conscious monitoring of responsibilities, deliberate regulation of emotions, decision making, supervisory attentional system, and verification of the fulfillment of objectives.

The main clinical implication arising from the present research has to do with the possibility of having an instrument that allows us to assess executive functions in real situations of university students, which will allow us to draw lines of performance of these skills in order to develop neuropsychological intervention plans and improve their performance, since the frontal structures finish maturing between 25 and 30 years of age and we are in time to help with these cognitive processes in the university student (Burnett et al., 2009).

The main limitation of this study, which must be considered, is the subjective nature involved in a self-report evaluation that will always generate a bias in the participant’s personal appreciation of their behavior; however, this point was taken care of by analyzing each case and applying the instrument, leaving out those that could present any inconsistency (Fenn et al., 2020).

Finally, having two Latin American cities in the research sample makes it possible to consider that similar realities are shared in Latin America at the level of executive functions, which motivates us to carry out new research involving new cities and the greatest amount possible of Latin American youth.

What arises from this study for future research involves several aspects, since it is in our interest to use the developed scale to analyze the relationship of executive functions with academic performance and behavior regulation in favor of university learning. Furthermore, it is in our best interest to generate intervention protocols to improve executive functioning and thus contribute towards an improved professional training of young university students in Latin America.

## Data availability statement

The raw data supporting the conclusions of this article will be made available by the authors, without undue reservation.

## Ethics statement

The studies involving human participants were reviewed and approved by Comité de Ética de la Pontificia Universidad Católica del Ecuador. Study code: 2019-58EO. The patients/participants provided their written informed consent to participate in this study.

## Author contributions

CR-G, VR, and MV: conceptualization, investigation, formal analysis, writing original draft, review and editing, and project administration. CR-G, NL, JC-C, PA-R, and MB-P: investigation, writing original draft, and formal analysis. CR-G and JC-C: investigation, formal analysis, and review and editing. All authors contributed to the article and approved the submitted version.

## Funding

This research was funded by Pontificia Universidad Católica del Ecuador and Universidad Tecnológica Indoamérica de Ecuador.

## Conflict of interest

The authors declare that the research was conducted in the absence of any commercial or financial relationships that could be construed as a potential conflict of interest.

## Publisher’s note

All claims expressed in this article are solely those of the authors and do not necessarily represent those of their affiliated organizations, or those of the publisher, the editors and the reviewers. Any product that may be evaluated in this article, or claim that may be made by its manufacturer, is not guaranteed or endorsed by the publisher.
