# Supplementary material for: Executive functions scale for university students: UEF-1
Source: Front Psychol. 2023 Jul 13;14:1192555. doi: 10.3389/fpsyg.2023.1192555 (PMC10372484; doi:10.3389/fpsyg.2023.1192555)
Supplement: Supplementary file 1 [file Table_1.docx]

# Annexes

**Executive functions scale for university settings UEF-1**

| **1** | **2** | **3** | **4** | **5** |
| --- | --- | --- | --- | --- |
| **Totally**  **disagree** | **Moderately**  **disagree** | **Don’t agree**  **or**  **disagree** | **Moderately**  **agree** | **Totally**  **agree** |

|  | Item in English | Item in Spanish | Executive function | Score | | | | |
| --- | --- | --- | --- | --- | --- | --- | --- | --- |
| 1 | It is easy to collect and leave my things organized when asked to do so. | Tengo facilidad para recoger y dejar ordenadas mis cosas cuando se me pide que lo haga. | Management of elements to solve tasks | 1 | 2 | 3 | 4 | 5 |
| 2 | I can finish a university assignment when it is very long. | Puedo terminar una tarea universitaria cuando es muy larga. | Conscious monitoring of responsibilities | 1 | 2 | 3 | 4 | 5 |
| 3 | I always act thinking and reflecting on the consequences of my actions. | Actúo siempre pensando y reflexionando las consecuencias de mis actos. | Conscious regulation of behavior | 1 | 2 | 3 | 4 | 5 |
| 4 | I properly regulate my emotions. | Regulateo adecuadamente mis emociones. | Deliberate regulation of emotions | 1 | 2 | 3 | 4 | 5 |
| 5 | I can make decisions independently. | Tengo la capacidad para tomar decisiones en forma independiente. | Decision making | 1 | 2 | 3 | 4 | 5 |
| 6 | I have my things in the right place and organized. | Tengo mis cosas en el lugar adecuado y en orden. | Management of elements to solve tasks | 1 | 2 | 3 | 4 | 5 |
| 7 | I have an easy time finding my materials by looking for them in my room or desk. | Tengo facilidad para encontrar rápidamente mis materiales al buscarlos en mi cuarto o escritorio. | Management of elements to solve tasks | 1 | 2 | 3 | 4 | 5 |
| 8 | I can complete college assignments independently and without help from others. | Puedo realizar las tareas universitarias de forma independiente y sin ayuda de los demás. | Conscious monitoring of responsibilities | 1 | 2 | 3 | 4 | 5 |
| 9 | I successfully complete my university assignments. | Logro realizar exitosamente mis trabajos de la universidad. | Conscious monitoring of responsibilities | 1 | 2 | 3 | 4 | 5 |
| 10 | I can concentrate well. | Tengo buena concentración. | Supervisory attentional system | 1 | 2 | 3 | 4 | 5 |
| 11 | I can be still and calm while I wait. | Puedo estar quieto/a y tranquilo/a mientras espero. | Conscious regulation of behavior | 1 | 2 | 3 | 4 | 5 |
| 12 | I can solve problems at university as well as in my personal life. | Tengo la capacidad para resolver problemas en la universidad como en mi vida personal. | Decision making | 1 | 2 | 3 | 4 | 5 |
| 13 | I focus on my university activities, leaving irrelevant things aside. | Me concentro en mis actividades universitarias, dejando de lado las cosas irrelevantes. | Supervisory attentional system | 1 | 2 | 3 | 4 | 5 |
| 14 | I can maintain my attention on an activity. | Soy capaz de mantener la atención en una actividad. | Supervisory attentional system | 1 | 2 | 3 | 4 | 5 |
| 15 | I can do my assignments without someone supervising me. | Puedo realizar mis trabajos sin que alguien me supervise. | Conscious monitoring of responsibilities | 1 | 2 | 3 | 4 | 5 |
| 16 | It is easy for me to behave appropriately in social gatherings. | Me es fácil comportarme adecuadamente en las reuniones sociales. | Conscious regulation of behavior | 1 | 2 | 3 | 4 | 5 |
| 17 | When someone asks me to, I can easily stop doing something that distracts me. | Cuando alguien me lo pide, puedo dejar con facilidad de hacer algo que me distrae. | Conscious regulation of behavior | 1 | 2 | 3 | 4 | 5 |
| 18 | I let others speak, without interrupting. | Dejo hablar a los demás, sin hacer interrupciones. | Conscious regulation of behavior | 1 | 2 | 3 | 4 | 5 |
| 19 | I can anticipate the consequences of my actions. | Puedo anticipar las consecuencias de mis actos. | Conscious regulation of behavior | 1 | 2 | 3 | 4 | 5 |
| 20 | I verify that my university assignments are well done and without errors, before giving them to the professor. | Verifico que mis tareas universitarias estén bien realizadas y sin errores, antes de presentarlas al profesor. | Verification of the fulfillment of objectives | 1 | 2 | 3 | 4 | 5 |
| 21 | I can make decisions without difficulty, even in the most complicated things. | Puedo tomar decisiones sin dificultad, incluso ante las cosas más complicadas. | Decision making | 1 | 2 | 3 | 4 | 5 |
| 22 | It is easy for me to concentrate on my college activities. | Me es fácil concentrarme en mis actividades universitarias. | Supervisory attentional system | 1 | 2 | 3 | 4 | 5 |
| 23 | I check the spelling and wording of my college assignments before I finish them. | Reviso la ortografía y redacción de mis tareas universitarias antes de finalizarlas. | Verification of the fulfillment of objectives | 1 | 2 | 3 | 4 | 5 |
| 24 | I remember to take home assignments, materials, or college papers. | Recuerdo llevar a casa las tareas, materiales o trabajos de la universidad. | Verification of the fulfillment of objectives | 1 | 2 | 3 | 4 | 5 |
| 25 | I can keep calm easily. | Mantengo la calma con facilidad. | Deliberate regulation of emotions | 1 | 2 | 3 | 4 | 5 |
| 26 | I pick up my mess without others having to do it for me. | Recojo mi desorden sin que otros lo hagan por mí. | Management of elements to solve tasks | 1 | 2 | 3 | 4 | 5 |
| 27 | I finish my college assignments on time. | Termino mis tareas universitarias a tiempo. | Conscious monitoring of responsibilities | 1 | 2 | 3 | 4 | 5 |
| 28 | I maintain good study habits. | Mantengo buenos hábitos de estudio. | Supervisory attentional system | 1 | 2 | 3 | 4 | 5 |
| 29 | My moods are stable. | Tengo un estado de ánimo estable. | Deliberate regulation of emotions | 1 | 2 | 3 | 4 | 5 |
| 30 | At the end of a university activity, I verify that I have achieved what I planned. | Al finalizar una actividad universitaria, verifico que haya logrado lo planificado. | Verification of the fulfillment of objectives | 1 | 2 | 3 | 4 | 5 |
| 31 | I can regulate my emotions. | Soy capaz de regulatear mis emociones. | Deliberate regulation of emotions | 1 | 2 | 3 | 4 | 5 |
